# *Yersinia pestis* genomes reveal plague in Britain 4000 years ago

**DOI:** 10.1038/s41467-023-38393-w

**Published:** 2023-05-30

**Authors:** Pooja Swali, Rick Schulting, Alexandre Gilardet, Monica Kelly, Kyriaki Anastasiadou, Isabelle Glocke, Jesse McCabe, Mia Williams, Tony Audsley, Louise Loe, Teresa Fernández-Crespo, Javier Ordoño, David Walker, Tom Clare, Geoff Cook, Ian Hodkinson, Mark Simpson, Stephen Read, Tom Davy, Marina Silva, Mateja Hajdinjak, Anders Bergström, Thomas Booth, Pontus Skoglund

**Affiliations:** 1 Ancient Genomics Laboratory, Francis Crick Institute, London, UK; 2 School of Archaeology, University of Oxford, Oxford, UK; 3 Independent Scholar, Wells, UK; 4 Oxford Archaeology, Osney Mead, Oxford UK; 5 Laboratoire Méditerranéen de Préhistoire Europe Afrique-UMR 7269, Centre National de la Recherche Scientifique, Aix-Marseille Université, Marseille, France; 6 Departamento de Prehistoria, Arqueología, Antropología Social y Ciencias y Técnicas Historiográficas, Universidad de Valladolid, Valladolid, Spain; 7 Department of Archaeology and New Technologies, Arkikus, Spain; 8 Wells & Mendip Museum, Wells, UK; 9 Levens Local History Group, Levens, Cumbria UK; 10 School of Biological and Environmental Sciences, Liverpool John Moores University, Liverpool, UK; 11 Department of Evolutionary Genetics and Department of Archaeogenetics, Max Planck Institute for Evolutionary Anthropology, Leipzig, Germany; 12 School of Biological Sciences, University of East Anglia, Norwich, UK

**Keywords:** Archaeology, Bacterial infection

## Abstract

Extinct lineages of *Yersinia pestis*, the causative agent of the plague, have been identified in several individuals from Eurasia between 5000 and 2500 years before present (BP). One of these, termed the ‘LNBA lineage’ (Late Neolithic and Bronze Age), has been suggested to have spread into Europe with human groups expanding from the Eurasian steppe. Here, we show that the LNBA plague was spread to Europe’s northwestern periphery by sequencing three *Yersinia pestis* genomes from Britain, all dating to ~4000 cal BP. Two individuals were from an unusual mass burial context in Charterhouse Warren, Somerset, and one individual was from a single burial under a ring cairn monument in Levens, Cumbria. To our knowledge, this represents the earliest evidence of LNBA plague in Britain documented to date. All three British *Yersinia pestis* genomes belong to a sublineage previously observed in Bronze Age individuals from Central Europe that had lost the putative virulence factor *yapC*. This sublineage is later found in Eastern Asia ~3200 cal BP. While the severity of the disease is currently unclear, the wide geographic distribution within a few centuries suggests substantial transmissibility.

## Abstract (web summary)

An extinct prehistoric plague lineage of *Yersinia pestis* has been documented from Central Europe to Asia during the Late Neolithic and Bronze Age. Here, Swali et al. show that this lineage spread to Europe’s northwestern periphery by sequencing three ~4000 year-old *Yersinia pestis* genomes from Britain.

## Introduction

*Yersinia pestis* is a zoonotic bacterium that can be transmitted via the bite of an infected flea vector, causing either bubonic or septicaemic plague, or via respiratory droplets through human-to-human contact causing pneumonic plague. Ancient DNA analysis has identified *Yersinia pestis* as the causative agent of not only historic epidemics such as the Justinianic plague^1–3^ and the Black Death^4,5^, but also identified previously unknown evidence of *Yersinia pestis* circulating in prehistory; Late Neolithic and Early Bronze Age (LNBA) plague has been found across Eurasia in the period between ~4700–2800 BP (years Before Present)^6–12^. The most frequently observed LNBA lineage lacks the *ymt* virulence factor (LNBA *ymt*-)^11,13^. The first known evidence of lineages with the *ymt* gene (LNBA *ymt*+) was found in a ~3800-BP-year-old individual (RT5) from Samara, Russia^9^ and a ~3300-BP-year-old individual (I2470) from Álava, Spain^11^.

The LNBA lineages were likely brought to Central and Western Europe ~4800 BP by human expansions originating in the Eurasian steppe^6,14,15^, and it has been hypothesised that these lineages contributed to the decline of certain Late Neolithic European societies^6,7^. However, it has been unclear how far LNBA plague spread throughout Europe, with the most westward finding of the LNBA *ymt-* lineage previously identified in present-day southern Germany ~3400 BP^11^. Human movements associated with the expansion of Bell Beaker cultures introduced steppe-derived ancestries to Britain and intensified links with continental Europe from ~4400 BP^16^, opening the possibility that Bronze Age groups from north-western Europe were also affected by LNBA lineages of *Yersinia pestis*, although this has not been observed directly until now.

Here, we show evidence of LNBA *Yersinia pestis* infections from two sites from different regions in Britain, suggesting that this lineage could have been widespread across Britain after the westward expansion of populations tracing ancestry to the Eurasian steppe. The LNBA *Yersinia pestis* lineage found in Britain belongs to a clade, which has undergone large losses of their genome, including the putative virulence factor *yapC*.

## Results

### Detection of *Yersinia pestis* in Bronze Age Britain

We sampled 34 individuals from two sites from the British Early Bronze Age—Charterhouse Warren and Levens Park—to screen for the presence of LNBA *Yersinia pestis* in Britain. We sampled teeth in ancient DNA cleanroom conditions and prepared double-indexed single-stranded DNA libraries, which we screened with 1.8 to 7.3 million paired-end reads each (Methods). We performed a metagenomic analysis in *Kraken 2*^17^ and identified individuals with a substantial excess of *k*-mers matching *Yersinia pestis* compared to the closely related species *Yersinia pseudotuberculosis* (Methods). This analysis identified two individuals (C10091/1233b and C10098/6265) out of 30 screened for pathogen DNA from Charterhouse Warren Farm in Somerset, United Kingdom (see Methods for further contextual details). The site is a mass burial assemblage of disarticulated human remains that were likely deposited together in a single event in a natural shaft. The two sampled teeth were derived from children aged 12 ± 3 yr and 10 ± 3 yr, respectively. The mandible associated with the C10098 tooth (6265/10-year-old child) has been directly radiocarbon dated to 4145–3910 cal BP (95.4% confidence; OxA-37840: 3685 ± 30 BP, calibrated in OxCal v4.4^18^ using IntCal20^19^), consistent with two previously published radiocarbon dates on human bones from this assemblage^20^. The other individual can be assumed to be of similar date.

In addition, our analysis identified *Yersinia pestis* in one of four individuals recovered from the Levens Park ring cairn monument in Levens, Cumbria, United Kingdom^21,22^ (see Methods for detailed archaeological context). One complete and three disturbed skeletons (Burials 1, 2, 3 and 4) were recovered from the monument and all have been directly dated to ca. 4300–3700 cal BP. We detected *Yersinia pestis* in a tooth from Burial 2 (C10928), a 35–45-year-old female recovered in a crouched position from a plank-lined grave (possibly a wooden coffin) and accompanied by Beaker pottery sherds. The skeleton has been directly dated to 4065–3780 cal BP (95.4% confidence, GU-51281: 3602 ± 33 BP^22^). Comparison of this radiocarbon date to the one obtained from Charterhouse C10098 suggests that we cannot reject the possibility that these two individuals were approximate contemporaries (*χ*^2^-test, df = 1, *T* = 2.746 (5% 3.841)).

**Fig. 1:**
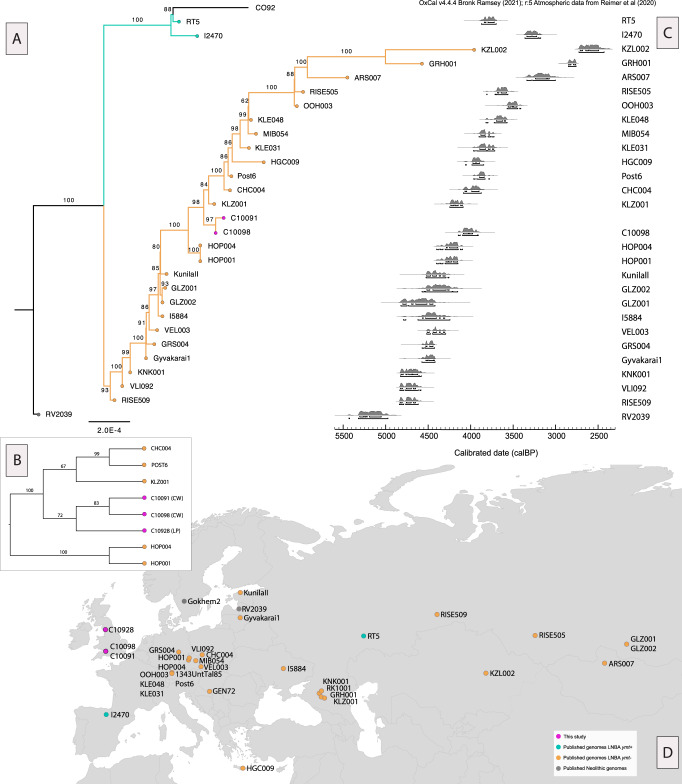
LNBA *Yersinia pestis* genomes. **A** Maximum likelihood phylogeny constructed in IQ-TREE v.1.6.12^29^ with 1000 bootstrap replicates using the TVMe+ASC substitution model, including previously published LNBA genomes (Supplementary Table 1) that passed the filtering criteria (Methods) as well as the two Charterhouse Warren genomes from this study. The phylogeny also includes the 1.ORI CO92 (NC_003143.1) reference genome and is rooted with IP32881 *Yersinia pseudotuberculosis* (not included in the figure). Numbers indicate the percentage of bootstrap replicates supporting that node. The orange branches denote the LNBA *ymt-* lineage (purple tips show individuals from this study, orange tips show published genomes that belong to the LNBA *ymt-* lineage) and the turquoise branches and tips denote the LNBA *ymt+* lineage. The grey tip node indicates the Neolithic strain. **B** Cladogram showing a maximum likelihood phylogeny that includes the lower coverage genome C10928 from Levens Park (LP), placing it close to the Charterhouse Warren (CW) strains, C10091 and C10098. **C** Chronology of *Yersinia pestis* genomes included in (A with the exception of C10091). Radiocarbon dates made directly on the individuals were calibrated in OxCal v4.4.4 using IntCal20^18, 19^ (Supplementary Table 3). **D** Map showing the distribution of LNBA and Neolithic *Yersinia pestis* strains. Points in purple show genomes newly sequenced in this study. Purple and orange points represent LNBA *ymt*- genomes, denoting the absence of the *ymt* gene and turquoise points represent LNBA *ymt*+ genomes (with *ymt* present). Points in grey show Neolithic strains of *Yersinia pestis*, which also lack the *ymt* virulence gene. Map made in R using ‘maps’ package and ggplot2.

These results from multiple sites demonstrate that *Yersinia pestis* had spread to Britain during the Bronze Age (Fig. 1A), but we note that the frequency of occurrence of *Yersinia pestis* remains unknown, and could be lower than the observed frequency in the two sites. The false-negative rate of *Yersinia pestis* detection in archaeological remains is unknown but likely high^23^, so we cannot exclude that other individuals at the sites were infected.

### Genome sequencing

**Fig. 2:**
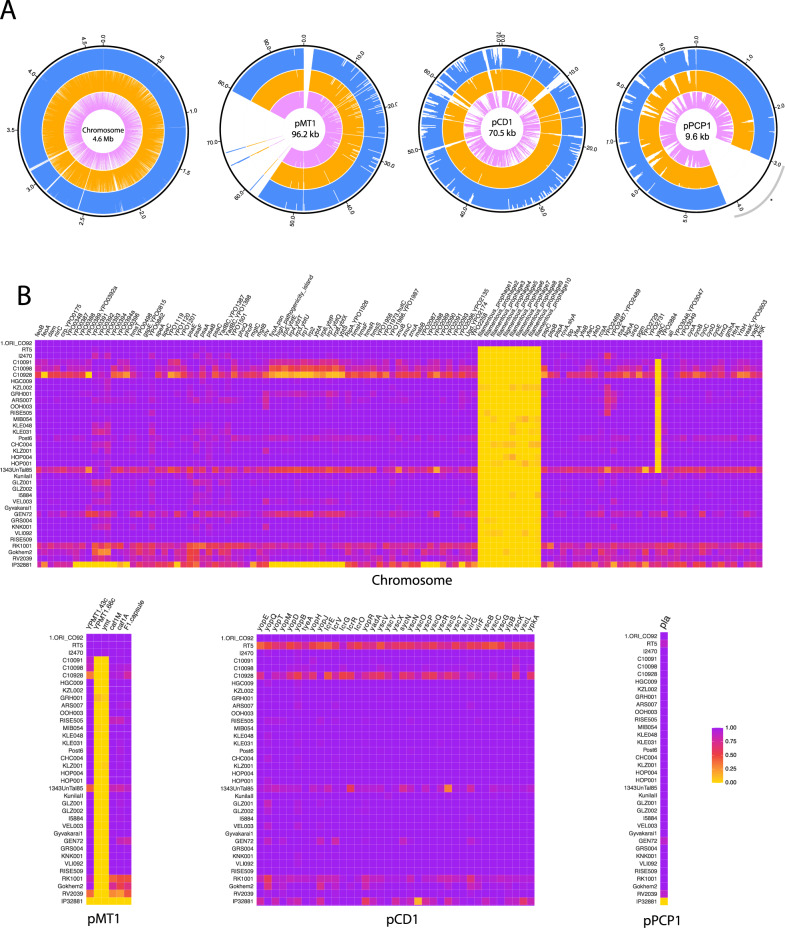
Coverage across *Yersinia pestis* chromosome, plasmids and associated virulence genes. **A** Circos plots of the coverage (MQ30) across the CO92 *Yersinia pestis* chromosome and plasmids pMT1, pCD1 and pPCP1 with blue representing C10091, orange C10098 and pink C10928. 500 bp windows for the chromosome, 100 bp windows for plasmids pMT1 and pCD1 and 10 bp windows for pPCP1 were used to calculate the average depth. Additionally, we manually masked the region between 3000–4200 bp on the pPCP1 plasmid (*) as in a previous study^59^. **B** Heatmap of inferred presence/absence of virulence-associated genes assessed by normalised depth-of-coverage across the *Yersinia pestis* chromosome and plasmids pCD1, pMT1 and pPCP1, with purple being 100% present, orange (midpoint) being 50% present and yellow being 0% present (Methods).

We used the Illumina NovaSeq platform to generate direct shotgun sequencing data of ~768 million and ~322 million read-pairs from individuals C10098 and C10091 from Charterhouse Warren, respectively (Table 1), after size selection to enrich for molecules at least 35 bp in length^24^ (Methods). The individual from Levens Park (C10928) was not amenable to shotgun sequencing but libraries from this individual, as well as from the two other individuals, underwent two rounds of hybridisation capture with an in-solution target enrichment approach using *Yersinia pestis* RNA baits (Daicel Arbor Biosciences). Merging data from the direct shotgun sequencing and the targeted enrichment experiments resulted in an average 8.4X, 6.1X and 3.3X coverage of the *Yersinia pestis* genome for C10091, C10098 and C10928, respectively when filtered with mapping quality of MQ1 (Table 1). All three genomes showed evidence of authenticity:^25^ postmortem damage, a decreasing number of observed sequences as a function of edit distance to the reference genome, a unimodal fragment length distribution, and a relatively even breadth of coverage across the genome (Supplementary Fig. 1 and Fig. 2A).

**Table 1:** Sequencing statistics for different libraries and experiments with a minimum mapping quality filter of q1 (MQ1)

	Total sequences, length > 35bp	Proportion Yersinia pestis (%)	Sequences aligned to Yersinia pestis after duplicate removal	Clonality (%)	Mean X-fold coverage (MQ1)	Breadth of coverage >1x (%) (MQ1)	Breadth of coverage >2x (%) (MQ1)	X fold coverage *pCD1* (MQ1)	X fold coverage *pMT1* (MQ1)	X fold coverage *pPCP1* (MQ1)
C10091 Capture	2,73,38,232	19.6	4,81,157	91.0	5.6	78.4	62.6	12.5	6.0	32.6
C10091 Shotgun	32,17,93,774	1.6	3,03,420	94.2	3.9	86.3	65.5	7.9	4.6	18.7
**C10091 final coverage**	**34,91,32,006**	3.0	**6,90,105**	93.5	**8.4**	**93.5**	**82.9**	**17.8**	**9.1**	**43.6**
C10098 Capture	1,56,97,965	36.7	3,36,045	94.2	4.6	70.2	53.0	12.3	5.1	30.3
C10098 Shotgun	76,77,47,954	0.02	87,634	49.1	1.7	56.2	22.1	2.9	1.6	7.2
**C10098 Final coverage**	**78,34,45,919**	0.8	**4,12,351**	93.1	**6.1**	**85.0**	**67.1**	**14.9**	**6.6**	**36.3**
C10928 Capture	19,49,92,184	4.3	2,08,793	97.5	3.3	61.3	41.7	5.5	3.2	23.7
C10928 Shotgun	46,76,025	0.002	88	3.3	0.001	0.0	0.0	0.0	0.0	0.01
**C10928 final coverage**	**19,96,68,209**	4.2	**2,08,881**	97.5	**3.3**	**61.3**	**41.7**	**5.5**	**3.2**	**23.7**

### Phylogenetic reconstruction

We aligned genomes from this study and previously published *Yersinia pestis* Neolithic and LNBA genomes^6–12,26^ (Supplementary Table 1) to the modern-day *Yersinia pestis* genome 1.ORI CO92 (NC_003143.1)^27^, and aligned the *Yersinia pseudotuberculosis* strain IP32881^28^ in the same way (Methods). We retained a subset of the genomes, which had a high degree of called sites given a per-site minimum sequence depth of three on all published genomes and a minimum depth of five for the in-house single-stranded library sequenced genomes (Methods). We performed phylogenetic inference with IQ-TREE^29^, assessed uncertainty with 1000 bootstrap replicates, and rooted the phylogeny with *Y. pseudotuberculosis* in FigTree. Our final phylogeny consisted of 27 ancient published genomes and both the Charterhouse Warren individuals (C10091 and C10098) from this study. We found that in the reconstructed phylogeny, the Charterhouse Warren *Yersinia pestis* genomes fall within the broader LNBA *ymt*- clade. In the asymmetrical clade of LNBA *ymt*- genomes, grouping broadly according to chronology^11^, we found that the British genomes are derived with respect to genomes from the Czech Republic (HOP001 and HOP004), dating to ~4300 cal BP and ~4250 cal BP, but basal to a genome from Russia, which dates to ~4200 cal BP (KLZ001)^11^. Indeed, the topology of the previously published genomes matches previously reconstructed phylogenies. We also placed the lower-coverage genome from Levens Park (C10928) in a reduced phylogeny that included the Charterhouse Warren genomes and high-coverage genomes from adjacent clades (Methods) (Fig. 1B), and found that it was likely closely related to those from Charterhouse Warren.

The two Charterhouse Warren genomes show 2 transversion mismatches out of 939,710 sites with a minimum sequence depth of 5 (a mismatch rate of 0.0002%). Both of these are due to apparent changes in the C10091 genome, and they are located adjacent to each other at positions 2227793 and 2227794 when aligned to the CO92 (NC_003143.1) reference genome. While BlastN searches of the NCBI nucleotide archive found that all sequences had *Yersinia pseudotuberculosis* and *Yersinia pestis* as the top match, the observed transversions were adjacent to an insertion (one sequence read further contained a deletion), and it therefore seems possible that they represent spurious alignment from a source uncharacterised in the database (Supplementary Fig. 2, Supplementary Table 2^30^).

### Deletions and functional elements

We searched for major deletions by assessing the sequence depth across the higher-coverage Charterhouse Warren genomes (Methods). We identified no new deletions larger than 1000 bp, but the previously identified ‘event 1’, the oldest deletion in LNBA strains, which resulted in the loss of a 36 kb region and was observed in multiple previous sequenced individuals^8,11^, is also observed in both the Charterhouse Warren and Levens Park genomes. The loss of this region resulted in the loss of the *yapC* virulence gene, which has been shown in vitro to mediate adhesion in cultured cells and could thus have enhanced the colonisation ability of *Yersinia pestis* during infection^31^.

We observe the presence and absence of other functional elements that are all congruent with the phylogenetic placement of the British *Yersinia pestis* genomes. We confirm the absence of the filamentous prophage, absent in all the published Neolithic and LNBA genomes, which is today mostly identified in 1.ORI strains^32^. The absence and presence of previously reported virulence factors on the British *Yersinia pestis* plasmids are all congruent with previously published LNBA *ymt*- plasmids (Fig. 2B). *UreD*, associated with increased flea toxicity^33^, *pde-2* which is involved in the downregulating mechanism for biofilm formation^34^ and *flhD*, the inactivation of which is associated with immune invasion^35^, all show variation consistent with no loss of function in the Charterhouse Warren genomes (Methods)^9^.

## Discussion

Our results reveal that the LNBA *ymt- Yersinia pestis* lineage was not confined to Bronze Age continental Europe, but had spread westward to Britain. We show that *Yersinia pestis* genomes in Britain had close affinities with Bronze Age genomes in present-day Germany, which may have been introduced by human expansions tracing back to the Eurasian steppe. Our results, alongside previously published genomes, thus show that in the period ~4700-2800 cal BP the LNBA *ymt*- strain of *Yersinia pestis* was widespread across Eurasia, from Britain to Eastern Asia. The distance between the Levens Park and Charterhouse Warren sites, in the northwest and southwest of England, respectively, and their statistically indistinguishable radiocarbon dates could suggest the widespread distribution of LNBA *ymt*- *Yersinia pestis* strains also across Britain.

Individuals from this study all lack the *ymt* virulence gene, which is known to have played an important role in flea-mediated transmission. The earliest known *Yersinia pestis* strain carrying the *ymt* gene has been found in the Samara region in the western part of the Eurasian steppe and dates to 3800 BP^9^. This is close in time to our two *Yersinia pestis* genomes from Britain, providing further evidence for differential contemporary frequencies of the *ymt* gene across an even wider geographic distance. The *ymt* gene, alongside the *hms* gene, enables survival and colonisation of the bacteria in the midgut and proventriculus of fleas, allowing for flea-mediated transmission^36–38^. However, research has also suggested an early-phase transmission model, which does not require the full extrinsic incubation period for flea-mediated transmission^39^. It has also been suggested that the *ymt* gene plays less of an important role in flea-mediated transmission for brown rat blood as opposed to blood from mice, humans, or black rats^40^.

In terms of the scale of deposition and evidence for extensive trauma, Charterhouse Warren is a rare site in the context of Early Bronze Age Europe^41^. It is clear that the individuals represented in the Charterhouse mass burial were subject to a non-normative form of funerary treatment, and it could be natural to suggest that this unusual treatment may have been a specific response to a local plague outbreak, much like the mass graves that appear across mediaeval Europe in response to the Black Death epidemic^42^. However, evidence of fatal trauma amongst the Charterhouse Warren assemblage (R. J. S., T. F-C., J. O., Christophe Snoeck, Fiona Brock, L. L., T. A., Don Walker, *in preparation)* makes it unlikely that this mass burial was due to a deadly outbreak of plague. Nevertheless, we cannot completely rule out a higher prevalence of plague amongst the remaining 28 individuals from this site as false negatives are possible, due to factors such as poor preservation of endogenous microbial DNA and variable pathogen load, making detection of ancient pathogens more difficult^23^. This raises the question–which we cannot answer at this point–of whether there may have been any connection between the disease and violent treatment of these individuals. At least one of the two child mandibles shows signs of perimortem trauma, although the disarticulated state of the remains means that potential trauma to the rest of the skeleton cannot be assessed. The presence of *Yersinia pestis* DNA in the two children suggests that they were either diseased when any trauma was inflicted on them, or alternatively that any trauma was inflicted after their death from the plague. The latter scenario cannot be dismissed altogether, although it is improbable given the rate of perimortem trauma observed in the assemblage as a whole, which suggests most or all individuals were killed during a specific violent event. By contrast, Burial 2 from Levens Park ring cairn represents a much more normative funerary rite for the period^22^. Further high-resolution sampling will be needed to answer these questions and better understand the transmission dynamics and functional evolution of *Yersinia pestis* in Britain and beyond.

## Methods

All radiocarbon dates were calibrated in OxCal 4.4 using the IntCal20 calibration curve^18,19^. There is no stable carbon and nitrogen isotopic evidence for any detectable input of marine or freshwater foods that would require a correction for reservoir effects.

### Charterhouse Warren: Archaeological context

Charterhouse Warren is a natural shaft in the limestone of the Mendip Hills, Somerset. Initial excavations in the 1970s were instigated in order to determine whether the shaft led to a cave system, as no archaeological remains from the site were known at that time. Excavators noted the presence of human and animal bones in a disarticulated state, some of which showed cutmarks and rodent gnawing^43^. The assemblage of human remains sampled for aDNA was encountered in a distinct layer at ca. 15 m depth, together with faunal remains and a small number of artefacts, including sherds of a Beaker vessel. At least 40 men, women and children are represented by fragmentary and disarticulated remains. Culturally and chronologically, the assemblage dates to the late Beaker period/Early Bronze Age, ca. 4150–3950 cal BP. This is a non-normative burial context for this period, which is dominated by single articulated burials associated with funerary monuments and cemeteries. Modifications found on a significant proportion of bones suggest that many, if not all, of these individuals had been subject to fatal perimortem trauma and subsequent processing before disarticulated bones were deposited together in the shaft in what is likely to have been a single event, though analysis of the assemblage and modelling of the radiocarbon dates is ongoing. While disarticulated remains are sometimes encountered in Bronze Age Britain, the scale of deposition at Charterhouse Warren is unique in a British context, suggesting that a very unusual event is represented. The evidence for blunt force perimortem trauma to a number of crania together with evidence for dismemberment suggests that this may relate to an episode of extreme violence.

### Levens Park: Archaeological context

The Levens Park ring cairn, consisting of a low kerbed mound below which were further rings of boulders or wall-like structures, in Levens, Cumbria, UK, was excavated between 1968 and 1971 by David Sturdy^21^. Examination of existing archival material by members of Levens Local History Group has produced a recent reassessment of the excavation, resulting in a more detailed understanding of the monument and its purpose^22^. We provide a general narrative of the site here but see Clare et al.^22^ for details on associated uncertainties. Four skeletons (Burials 1, 2, 3 and 4) were recovered from the monument and represent two separate phases of burial activity. Burial 4 comprised an unaccompanied crouched burial of a 25–45-year-old male covered with a large boulder but no associated artefacts. The skeleton has been radiocarbon dated to 4229-3976 cal BP (95% confidence, 3731 ± 34 BP, GU-51283), significantly earlier than dates from the other three skeletons (R_Combine *χ*^2^ Test in OxCal 4.4: df = 3, *T* = 11.2 (5%, 11.8))^18,19^. Burials 1, 2 and 3 were recovered from a larger adjacent structure within the monument. Their radiocarbon dates were statistically indistinguishable, suggesting they could have died around the same time or within a few decades of one another (R_Combine *χ*^2^ Test in OxCal 4.4: df = 2, *T* = 0.7 (5%, 6.0));^18,19^ Burial 2, which is thought to be the primary interment in this phase, owing to its position in the centre of the new structure, was a 35–45-year-old female recovered in a crouched position from a plank-lined grave (possibly a wooden coffin) and accompanied by sherds of Beaker pottery. The skeleton has been directly dated to 4065–3780 cal BP (95% confidence, 3602 ± 33 BP, GU-51281). Burial 1, a 17–35-year-old female dating to 4080–3840 cal BP, 95% confidence, 3626 ± 33 BP, GU-51280), and Burial 3, a probable adult female dating to 3982–3879 cal BP (95% confidence, 3587 ± 33 BP, GU-51282), were recovered from the cairn matrix and their original character and associations are unclear. Strontium and oxygen stable isotope analyses of Burials 1, 3 and 4 are consistent with them having grown up locally, although the results would also be consistent with an origin in other parts of Britain, likewise Burial 2.

### Sampling, DNA extraction and library preparation

Samples were processed at The Francis Crick Institute in a dedicated cleanroom facility. We used an EV410-230 EMAX Evolution Dentistry drill to clean the surface of the teeth and sampled both the cementum and multiple fractions of the dentine, resulting in 7–25 mg of powder from the dentine. Dentine powders were then lysed with 300 µl (<10 mg of powder), 600 µl (10–25 mg) or 1000 µl (>25 mg of powder) of lysis buffer (0.5 EDTA pH 8.0, 0.05% Tween-20, 0.25 mg/ml Proteinase K^44^) and incubated overnight at 37 °C. Lysates were centrifuged for 2 min at maximum speed of 16,400 × *g* (13,200 rpm) in a table centrifuge and 140 µl of the lysate was transferred into FluidX tubes for automated extraction on an Agilent Bravo Workstation^45^. Extracts were turned into single-stranded DNA libraries^24^, then double-indexed^46^ and underwent paired-end sequencing on an Illumina HiSeq4000 or NextSeq500 platform resulting in 1.8 to 7.3 million read-pairs per sample for initial screening. All samples were processed alongside negative lysate and extraction controls as well as positive and negative library controls.

### Targeted enrichment

Following initial pathogen detection, libraries were taken forward for target enrichment using *Yersinia pestis* baits predesigned by myBaits Arbor Biosciences, following myBaits custom RNA seq v5.1 (March 2021) High Sensitivity protocol^47^. We used 7 μl of the initial library for two rounds of hybridisation at 55 °C with 23-h overnight incubation and 20 PCR cycles, followed by a heteroduplex removal using a one-cycle PCR and MinElute Purification (Qiagen) cleanup. We sequenced each library with a 2 × 100 paired-end read configuration on the Illumina MiSeq and NovaSeq platforms (Table 1).

### Size selection for shotgun sequencing

Before larger-scale direct shotgun sequencing, fragments shorter than 35 bp and longer than 150 bp were removed from the libraries, as in Gansauge et al.^24^. Specifically, 100 ng of the initial library was biotinylated and streptavidin beads were used to isolate the non-biotinylated strand and obtain a single-stranded library. These samples (pooled with 3 others) were then loaded on a denaturing polyacrylamide gel along with 35 bp and 150 bp insert markers, and fragments within the desired sequence length were physically excised and eluted from the gel, after overnight incubation. The resulting size-selected libraries were further amplified and sequenced on the Illumina NovaSeq (Table 1).

### Bioinformatic processing

Samples were processed via the *nf-core/eager v2* pipeline^48^. First, adapters were removed, paired-end reads were merged and bases with a quality below 20 were trimmed using *AdapterRemoval v2*^49^ with –trimns –trimqualities –collapse –minadapteroverlap 1 and –preserve5p. Merged reads with a minimum length of 35 bp were mapped to the hs37d5 human reference genome with *Burrows-Wheeler Aligner* (*BWA-0.7.17 aln*)^50^ using the following parameters “-l 16500 -n 0.01”^51,52^.

### Metagenomic screening and authentication

We analysed sequences that did not align successfully to the human genome using *Kraken 2*^17^ and identified individuals as putatively positive for *Yersinia pestis* by assessing the number of observed *k*-mers (sequence matches) to *Yersinia pestis* compared to *Yersinia pseudotuberculosis*. These libraries were subsequently aligned to the CO92 *Yersinia pestis* reference genome (NC_003143.1) and the CO92 plasmids, pMT1 (NC_003134.1), pCD1 (NC_003131.1) and pPCP1 (NC_003132.1) using the BWA aln^50^ parameters “-l 16500 -n 0.01 -o 2”. Duplicates were removed by keeping only the first sequence out of any set of sequences with the same start position and length (https://github.com/pontussk/samremovedup). We assessed the authenticity of the final set of sequences using the following criteria:^25^ the observation of postmortem damage, with the number of sequences being negatively correlated with edit distance from the reference genome, and a unimodal fragment length distribution via *DamageProfiler*^53^. Additionally, we used *SAMTools v1.3.1* depth^54^ to confirm an even breadth of coverage across the reference genome. Screening libraries that passed these authentication criteria, were taken forward for further shotgun sequencing and target enrichment. For the final BAM files, we merged shotgun and target enriched BAM files using samtools merge resulting in a final coverage of 8.4x, 6.1x and 3.3x coverage for C10091, C10098 and C10928, respectively when filtered on a minimum mapping quality of q1 (MQ1) (Table 1, Fig. 2A, Supplementary Fig. 1).

### Analysis of functional elements and virulence factors

We used previously identified virulence genes^11^ on the *Yersinia pestis* chromosome, pCD1, pMT1 and pPCP1 plasmids, and analysed the coverage of these genes with *BEDTools* v2.29.2^55^ (Fig. 2A). The *ggplot2*^56^ package in R was then used to make a coloured heatmap of the percentage of positions in each virulence gene covered by at least one sequence. Additionally, we used *BEDTools* v2.29.2^55^ genomecov to assess the missingness across the genome and identify regions that were larger than 1 kb in the two Charterhouse individuals. Some differences in coverage across the *Yersinia pestis* chromosome between the pre-designed Arbor RNA capture baits compared to shotgun data can be observed in direct comparisons, most likely due to preferential capture of some parts of the chromosome over others with the bait set (Supplementary Fig. 3). However, we see no evidence that this could lead to conclusions about major deletions or loss of function. We manually inspected the *ureD, pde-2 and flhD* genes to identify the presence of indels and SNPs specific to these genes, to assess whether they were functional or not in the Charterhouse Warren genomes^9^. However, the coverage for C10928 was too low to assess these indels.

### Mismatches between the two Charterhouse genomes

We compared the two Charterhouse Warren genomes to identify transversion SNPs at differing base-fold coverages (Supplementary Fig. 2, Supplementary Table 2) after filtering for a mapping and base quality of 30 using *samtools calmd*^54^ and removing heterozygous sites using an in-house script (https://github.com/pontussk/mpileup_mismatch_pathogen.py).

### Phylogenetic reconstruction

Previously published data from Bronze age and Neolithic plague genomes and the IP32881 strain of *Yersinia pseudotuberculosis* were downloaded from the European Nucleotide Archive (Supplementary Table 1) and processed using the same parameters as data generated in this study, described above (Bioinformatic processing) with the exception of duplicate removal, which removed duplicates with the same start position regardless of length to adjust for single-end sequencing in published data.

After duplicate read removal, we used an in-house script (https://github.com/pontussk/mpileup2consensus.py) to keep only homozygous sites with a base quality score of Q30, thus excluding any heterozygous sites and small (shorter than a sequence read length) insertions or deletions and a maximum edit distance of 90% using *samtools calmd*^54^. We then filtered all sites to retain only sites with a minimum base coverage of 3-fold to obtain a consensus sequence for all published genomes and a 5-fold coverage for the Charterhouse Warren genomes.

Consensus sequences were then filtered to keep only sites that were polymorphic within the set of sequences used for input in the phylogeny, removing all transitions to remove effects of cytosine deamination in the ancient DNA sequences. For the main phylogeny (Fig. 1A) we excluded individuals with more than 70% missingness across the genome and removed sites that were missing in more than 20% of the remaining individuals, resulting in 2306 transversion SNPs that were taken forward to IQ-TREE v.1.6.12^29^. We implemented model testing using the ModelFinder in IQ-TREE^57^, which suggested a TVMe+ASC as the best-fit model according to the Akaike information criterion for both phylogenies in Fig. 1A and Fig. 1B. We implemented 1000 bootstrap replicates for the phylogeny in Fig. 1A and rooted the maximum likelihood phylogeny in *FigTree*^58^, with the *Yersinia pseudotuberculosis* outgroup (IP32881) (the outgroup is not shown in the figure to increase the visibility of the LBNA clades in Fig. 1A).

Due to its low coverage, a separate phylogenetic inference was used to position the Levens Park genome, C10928. Here, we took the filtered consensus sequences of the LNBA strains, which were topologically close to the monophyletic Charterhouse Warren (C10091 and C10098) group, and filtered them to a minimum base coverage of 3, keeping transversions only and sites present in at least 70% of the genomes (regardless of missingness across the genomes). This resulted in 104 SNPs taken forward to IQ-TREE, using a TVMe+ASC model with 100 bootstrap replicates. The maximum likelihood phylogeny was rooted in *FigTree*^58^ based on Fig. 1A and transformed into the cladogram in Fig. 1B.

### Reporting summary

Further information on research design is available in the Nature Portfolio Reporting Summary linked to this article.

## Supplementary information

Supplementary Information

Reporting Summary

## Data Availability

Sequencing data generated in this study have been deposited in the European Nucleotide Archive (ENA) at EMBL-EBI under accession number PRJEB61230.

## Code availability

Code used in this study is made available at https://github.com/pontussk/.
