# Supplementary material for: Yersinia pestis genomes reveal plague in Britain 4000 years ago
Source: Nat Commun. 2023 May 30;14:2930. doi: 10.1038/s41467-023-38393-w (PMC10229654; doi:10.1038/s41467-023-38393-w)
Supplement: Supplementary file 1 — Supplementary Information [file 41467_2023_38393_MOESM1_ESM.pdf]

Supplementary Material

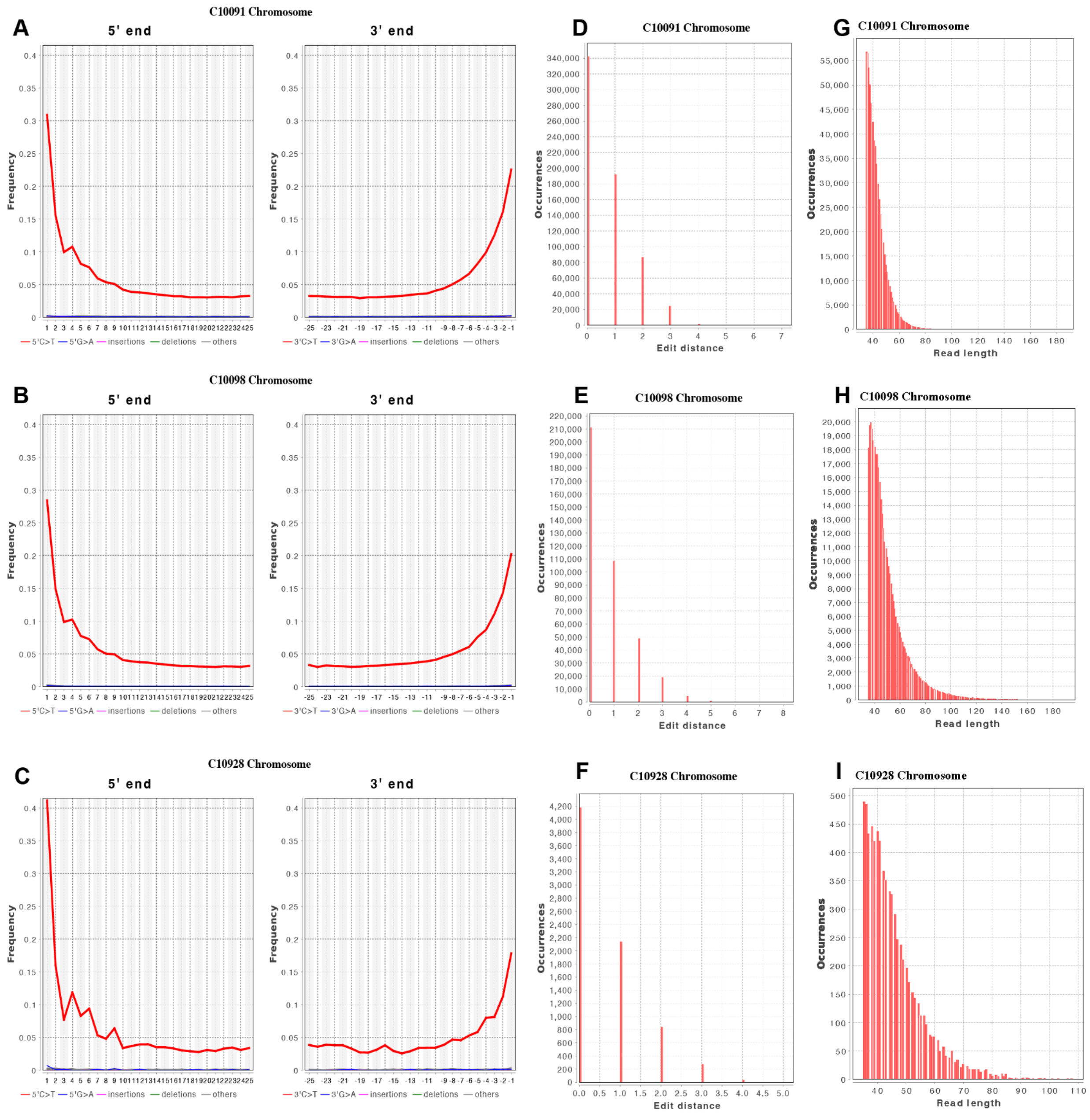

Supplementary Figure 1. Authentication metrics for *Yersinia pestis* genomes from Charterhouse Warren (C10091 and C10098) and Levens Park (C10928) chromosomes when aligned to CO92 reference genome[1].

A-C) Nucleotide misincorporations resulting from cytosine deamination. **D-F**) Number of sequences with an edit distance of 1 to 4 from the *Yersinia pestis* reference genome. **G-I**) Fragment length distribution. All *Yersinia pestis* genomes in this study were authenticated using *DamageProfiler*[2].

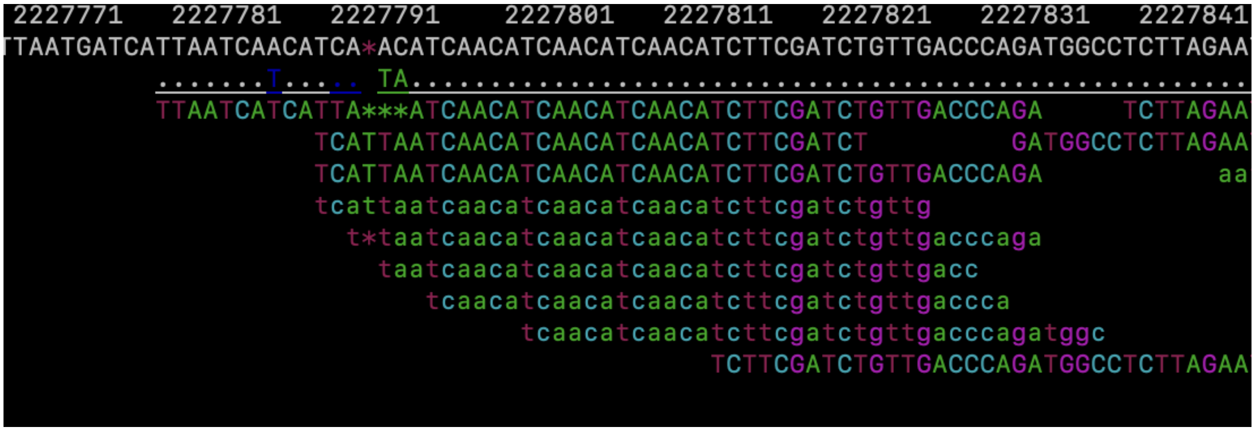

Supplementary Figure 2. Visual alignment of C10091 sequence alignment. SAMtools *v1.3.1* tview[3] alignment view of C10091 showing positions 2227793 and 2227794 when aligned to the CO92[1] (NC_003143.1) reference sequence.

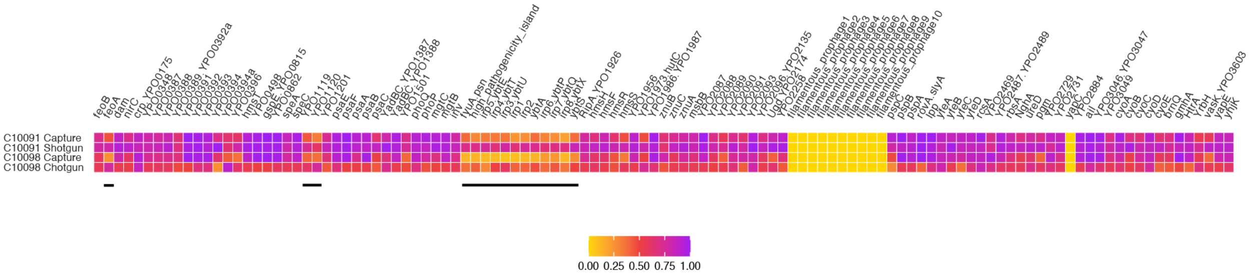

Supplementary Figure 3. Comparison of the coverage of virulence genes on the *Yersinia pestis* chromosome for captured (targeted enrichment) and shotgun-sequenced libraries. Heatmap of inferred presence/absence of virulence-associated genes assessed by normalised depth-of-coverage across the *Yersinia pestis* chromosome separated by whether libraries were captured or shotgun sequenced. In the captured libraries, some regions (underlined in black) have lower coverage in captured libraries than in the shotgun libraries. Given this capture-specific pattern, there is no evidence of novel deletions in the Levens Park genome, which is primarily reconstructed with capture (**Figure 2B**). Libraries were captured using myBaits Arbor Biosciences, following myBaits custom RNA seq v5.1 (March 2021) High Sensitivity protocol.

Supplementary Table 1. Published ancient *Yersinia pestis* genomes used in this study.

Yersinia pestis genome	Lineage	Publication
Gokhem2	Neolithic	Rascovan et al. 2019[4]/ Skoglund et al. 2014[5]
RV2039	Neolithic	Susat et al. 2021[6]
I2470	LNBA *ymt+*	Andrades Valtueña et al. 2022[7]
RT5	LNBA *ymt+*	Spyrou et al. 2018[8]
GEN72	LNBA *ymt-*	[9]et al. 2017
Gyvakarai1	LNBA *ymt-*	Andrades Valtueña et al. 2017
Post6	LNBA *ymt-*	Andrades Valtueña et al. 2017
1343UnTal85	LNBA *ymt-*	Andrades Valtueña et al. 2017
RK1001	LNBA *ymt-*	Andrades Valtueña et al. 2017
KunilaII	LNBA *ymt-*	Andrades Valtueña et al. 2017
OOH003	LNBA *ymt-*	Andrades Valtueña et al. 2022[7]
I5884	LNBA *ymt-*	Andrades Valtueña et al. 2022[7]
VLI092	LNBA *ymt-*	Andrades Valtueña et al. 2022[7]
KNK001	LNBA *ymt-*	Andrades Valtueña et al. 2022[7]
GRS004	LNBA *ymt-*	Andrades Valtueña et al. 2022[7]
HOP001	LNBA *ymt-*	Andrades Valtueña et al. 2022[7]
HOP004	LNBA *ymt-*	Andrades Valtueña et al. 2022[7]
KLZ001	LNBA *ymt-*	Andrades Valtueña et al. 2022[7]
CHC004	LNBA *ymt-*	Andrades Valtueña et al. 2022[7]
KLE031	LNBA *ymt-*	Andrades Valtueña et al. 2022[7]
MIB054	LNBA *ymt-*	Andrades Valtueña et al. 2022[7]
KLE048	LNBA *ymt-*	Andrades Valtueña et al. 2022[7]
ARS007	LNBA *ymt-*	Andrades Valtueña et al. 2022[7]
KZL002	LNBA *ymt-*	Andrades Valtueña et al. 2022[7]
GRH001	LNBA *ymt-*	Andrades Valtueña et al. 2022[7]
VEL003	LNBA *ymt-*	Andrades Valtueña et al. 2022[7]
HGC009	LNBA *ymt-*	Neumann et al. 2022[7]
RISE509	LNBA *ymt-*	Rasmussen et al. 2015[10]
RISE505	LNBA *ymt-*	Rasmussen et al. 2015[10]
C10091, Charterhouse Warren	LNBA *ymt-*	This Study
C10098, Charterhouse Warren	LNBA *ymt-*	This Study
C10928, Levens Park	LNBA *ymt-*	This Study
GZL002	LNBA *ymt-*	Yu et al. 2020[11]
GZL001	LNBA *ymt-*	Yu et al. 2020[11]
C092*, Yersinia Pestis*	1.ORI	Parkhill et al. 2001[1]
IP32881, *Yersinia pseudotuberculosis*	Outgroup *Yersinia Pseudotuberculosis*	Reuter et al. 2014[12]

Supplementary Table 2. Top 4 BlastN hits of the sequences in C10091 overlapping positions 2227793 and 2227794 when aligned to the CO92 (NC_003143.1) reference sequence.

Sequence in C10091 overlapping position 2227793 and 2227794	Top hits	BlastN top 4 hits	Max Score	Total Score	Query cover	E value	Percentage identity	Accession Length	Accession
TTAATCATCATTA*** ATCAACATCAACAT CAACATCTTCGATC TGTTGACCCAGA	1	*Yersinia pseudotuberculosis*	93.5	93.5	100%	2.00E-15	98.11%	4367018	CP044064.1
	2	*Yersinia pseudotuberculosis*	93.5	93.5	100%	2.00E-15	98.11%	4610200	LR134373.1
	3	*Yersinia pestis subsp. pestis*	93.5	93.5	100%	2.00E-15	98.11%	828	MF662143.1
	4	*Yersinia pseudotuberculosis*	93.5	93.5	100%	2.00E-15	98.11%	4728337	CP009786.1
TCATTAATCAACATC AACATCAACATCTT CGATCT	1	*Yersinia pseudotuberculosis*	65.8	65.8	100%	7.00E-08	100.00%	4367018	CP044064.1
	2	*Yersinia pseudotuberculosis*	65.8	65.8	100%	7.00E-08	100.00%	4610200	LR134373.1
	3	*Yersinia pseudotuberculosis*	65.8	65.8	100%	7.00E-08	100.00%	4938759	LR134306.1
	4	*Yersinia pestis subsp. pestis*	65.8	65.8	100%	7.00E-08	100.00%	828	MF662143.1
TCATTAATCAACATC AACATCAACATCTT CGATCTGTTGACC CAGA	1	*Yersinia pseudotuberculosis*	86.1	86.1	100%	2.00E-13	100.00%	4367018	CP044064.1
	2	*Yersinia pseudotuberculosis*	86.1	86.1	100%	2.00E-13	100.00%	4610200	LR134373.1
	3	*Yersinia pestis subsp. pestis*	86.1	86.1	100%	2.00E-13	100.00%	828	MF662143.1
	4	*Yersinia pseudotuberculosis*	86.1	86.1	100%	2.00E-13	100.00%	4728337	CP009786.1
tcattaatcaacatcaaca tcaacatcttcgatctgttg	1	*Yersinia pseudotuberculosis*	73.1	73.1	100%	8.00E-10	100.00%	4367018	CP044064.1
	2	*Yersinia pseudotuberculosis*	73.1	73.1	100%	8.00E-10	100.00%	4610200	LR134373.1
	3	*Yersinia pseudotuberculosis*	73.1	73.1	100%	8.00E-10	100.00%	4938759	LR134306.1
	4	*Yersinia pestis subsp. pestis*	73.1	73.1	100%	8.00E-10	100.00%	828	MF662143.1
t*taatcaacatcaacatc aacatcttcgatctgttga cccaga	1	*Yersinia pseudotuberculosis*	80.5	80.5	100%	6.00E-12	100.00%	4367018	CP044064.1
	2	*Yersinia pseudotuberculosis*	80.5	80.5	100%	6.00E-12	100.00%	4610200	LR134373.1
	3	*Yersinia pestis subsp. pestis*	80.5	80.5	100%	6.00E-12	100.00%	828	MF662143.1
	4	*Yersinia pseudotuberculosis*	80.5	80.5	100%	6.00E-12	100.00%	4728337	CP009786.1
taatcaacatcaacatca acatcttcgatctgttgac c	1	*Yersinia pseudotuberculosis*	71.3	71.3	100%	2.00E-09	100.00%	4367018	CP044064.1
	2	*Yersinia pseudotuberculosis*	71.3	71.3	100%	2.00E-09	100.00%	4610200	LR134373.1
	3	*Yersinia pseudotuberculosis*	71.3	71.3	100%	2.00E-09	100.00%	4938759	LR134306.1
	4	*Yersinia pestis subsp. pestis*	71.3	71.3	100%	2.00E-09	100.00%	828	MF662143.1

Supplementary Table 3. Radiocarbon dates of individuals included in Figure 1B. Calibrated in OxCal 4.4[13] using IntCal20[14]. GLZ001 and GLZ002 have been corrected for a freshwater reservoir effect following Schulting et al. (2022)[15]; original dates are given under Source. RV2039 have been corrected for a freshwater reservoir effect following Brinker et al. (2020)[16]; original date given under Source. I5884 has been estimated by Andrades Valtueña et al. (2022) to be too old using BEAST v2.6.6, and has been corrected here by -200 14C years, and its error term increased to reflect uncertainty in the correction.

Site	Country	Individual	Lab/sample code	14C yr	±	cal BP (95.4%)		Source
Mikhailovsky II	Russia	RT5	MAMS-29430	3517	27	3873	3699	Spyrou et al. 2018
El Sotillo	Spain	I2470	Beta-299307	3060	30	3361	3176	Andrades Valtueña et al. 2022
Kyzyl	Kazakhstan	KZL002	UBA-25474	2491	33	2725	2430	Andrades Valtueña et al. 2022
Grushevskoe	Russia	GRH001	MAMS-47775	2707	20	2850	2760	Andrades Valtueña et al. 2022
Arbulag soum	Mongolia	ARS007	NSF-Arizona	2991	48	3338	3003	Andrades Valtueña et al. 2022
Kytmanovo	Russia	RISE505	OxA-31216	3391	27	3811	3561	Rasmussen et al. 2015
Oberottmarshausen	Germany	OOH003	MAMS-21543	3283	32	3571	3410	Andrades Valtueña et al. 2022
Kleinaitingen	Germany	KLE048	MAMS-21595	3417	27	3819	3572	Andrades Valtueña et al. 2022
Mikulovice	Czech Republic	MIB054	MAMS-30479	3550	19	3901	3725	Andrades Valtueña et al. 2022
Kleinaitingen	Germany	KLE031	MAMS-21584	3548	34	3965	3716	Andrades Valtueña et al. 2022
Hagios Charalambos	Crete	HGC009	MAMS-45081	3621	22	4061	3847	Neumann et al. 2022
Postillionstrasse	Germany	Post6	MAMS-18955	3574	19	3964	3779	Andrades Valtueña et al. 2017
Chociwel	Poland	CHC004	Ua-44034	3645	33	4086	3875	Andrades Valtueña et al. 2022
Kaluzhnyy 1	Russia	KLZ001	MAMS-45952	3793	25	4246	4088	Andrades Valtueña et al. 2022
Charterhouse Warren	England	C10098	OxA-37840	3685	30	4145	3910	This Study
Hostivice-Palouky	Czech Republic	HOP004	MAMS-38921	3826	27	4400	4096	Andrades Valtueña et al. 2022
Hostivice-Palouky	Czech Republic	HOP001	MAMS-30798	3837	24	4401	4150	Andrades Valtueña et al. 2022
Kunilall	Estonia	Kunilall	Poz-10825	3960	40	4524	4256	Andrades Valtueña et al. 2017
Glazkovskoe	Russia	GLZ002	GLZ002	4077	68	4571	4153	Yu et al. 2020 (4369 ± 22)
Glazkovskoe	Russia	GLZ001	GLZ001	3939	68	4821	4420	Yu et al. 2020 (4319 ± 22)
Dereivka I	Ukraine	I5884	PSUAMS-282, PSUAMS-7830	3989	60	4790	4244	Andrades Valtueña et al. 2022; Patterson et al. 2022 (4189 ± 17)
Velešovice	Czech Republic	VEL003	MAMS-34184	3951	26	4518	4294	Andrades Valtueña et al. 2022
Großstorkwitz	Germany	GRS004	Hd-21977	4039	17	4571	4425	Andrades Valtueña et al. 2022
Gyvakarai	Lithuania	Gyvakarai1	Poz-61584	4030	30	4574	4418	Andrades Valtueña et al. 2017
Krasnogvardeyskoe	Russia	KNK001	MAMS-45959	4158	26	4827	4580	Andrades Valtueña et al. 2022
Vlineves	Czech Republic	VLI092	MAMS-45801	4176	26	4834	4585	Andrades Valtueña et al. 2022
Bateni, Afanasievo	Russia	RISE509	OxA-31221	4186	27	4838	4617	Rasmussen et al. 2015
Riņņukalns	Latvia	RV2039	KIA-46462	4519	57	5432	4973	Susat et al 2021; Brinker et al. 2020 (5049 ± 33)

Supplementary References

1. Parkhill, J. *et al.* Genome sequence of Yersinia pestis, the causative agent of plague. *Nature* **413**, 523–527 (2001).
2. Neukamm, J., Peltzer, A. & Nieselt, K. DamageProfiler: Fast damage pattern calculation for ancient DNA. *Bioinformatics* (2021) doi:10.1093/bioinformatics/btab190.
3. Li, H. & Durbin, R. Fast and accurate short read alignment with Burrows-Wheeler transform. *Bioinformatics* **25**, 1754–1760 (2009).
4. Rascovan, N. *et al.* Emergence and Spread of Basal Lineages of Yersinia pestis during the Neolithic Decline. *Cell* **176**, 295–305.e10 (2019).
5. Skoglund, P. *et al.* Genomic diversity and admixture differs for Stone-Age Scandinavian foragers and farmers. *Science* **344**, 747–750 (2014).
6. Susat, J. *et al.* A 5,000-year-old hunter-gatherer already plagued by Yersinia pestis. *Cell Rep.* **35**, 109278 (2021).
7. Andrades Valtueña, A. *et al.* Stone Age Yersinia pestis genomes shed light on the early evolution, diversity, and ecology of plague. *Proc. Natl. Acad. Sci. U. S. A.* **119**, e2116722119 (2022).
8. Spyrou, M. A. *et al.* Analysis of 3800-year-old Yersinia pestis genomes suggests Bronze Age origin for bubonic plague. *Nat. Commun.* **9**, 2234 (2018).
9. Andrades Valtueña, A. *et al.* The Stone Age Plague and Its Persistence in Eurasia. *Curr. Biol.* **27**, 3683–3691.e8 (2017).
10. Rasmussen, S. *et al.* Early divergent strains of Yersinia pestis in Eurasia 5,000 years ago. *Cell* **163**, 571–582 (2015).
11. Yu, H. *et al.* Paleolithic to Bronze Age Siberians Reveal Connections with First Americans and across Eurasia. *Cell* **181**, 1232–1245.e20 (2020).
12. Reuter, S. *et al.* Parallel independent evolution of pathogenicity within the genus Yersinia. *Proc. Natl. Acad. Sci. U. S. A.* **111**, 6768–6773 (2014).
13. Bronk Ramsey, C. Development of the Radiocarbon Calibration Program. *Radiocarbon* **43**, 355–363 (2001).
14. Reimer, P. J. *et al.* The IntCal20 Northern Hemisphere Radiocarbon Age Calibration Curve (0–55 cal kBP). *Radiocarbon* **62**, 725–757 (2020).
15. Schulting, R. J. *et al.* Freshwater reservoir effects in Cis-Baikal: An overview. *Archaeological Research in Asia* **29**, 100324 (2022).
16. Brinker, U. *et al.* Two burials in a unique freshwater shell midden: insights into transformations of Stone Age hunter-fisher daily life in Latvia. *Archaeol. Anthropol. Sci.* **12**, 97 (2020).
