# Supplementary material for: Yersinia pestis genomes reveal plague in Britain 4000 years ago
Source: Nat Commun. 2023 May 30;14:2930. doi: 10.1038/s41467-023-38393-w (PMC10229654; doi:10.1038/s41467-023-38393-w)
Supplement: Supplementary file 2 — Reporting Summary [file 41467_2023_38393_MOESM2_ESM.pdf]

nature portfolio

Corresponding author(s): Pooja Swali, Pontus Skoglund

Last updated by author(s): Mar 29, 2023

Reporting Summary

Nature Portfolio wishes to improve the reproducibility of the work that we publish. This form provides structure for consistency and transparency in reporting. For further information on Nature Portfolio policies, see our Editorial Policies and the Editorial Policy Checklist.

Statistics

For all statistical analyses, confirm that the following items are present in the figure legend, table legend, main text, or Methods section.

n/a	Confirmed	
☐	☒	The exact sample size (*n*) for each experimental group/condition, given as a discrete number and unit of measurement
☒	☐	A statement on whether measurements were taken from distinct samples or whether the same sample was measured repeatedly
☒	☐	The statistical test(s) used AND whether they are one- or two-sided<br>*Only common tests should be described solely by name; describe more complex techniques in the Methods section.*
☒	☐	A description of all covariates tested
☒	☐	A description of any assumptions or corrections, such as tests of normality and adjustment for multiple comparisons
☐	☒	A full description of the statistical parameters including central tendency (e.g. means) or other basic estimates (e.g. regression coefficient) AND variation (e.g. standard deviation) or associated estimates of uncertainty (e.g. confidence intervals)
☒	☐	For null hypothesis testing, the test statistic (e.g. *F*, *t*, *r*) with confidence intervals, effect sizes, degrees of freedom and *P* value noted<br>*Give P values as exact values whenever suitable.*
☒	☐	For Bayesian analysis, information on the choice of priors and Markov chain Monte Carlo settings
☒	☐	For hierarchical and complex designs, identification of the appropriate level for tests and full reporting of outcomes
☒	☐	Estimates of effect sizes (e.g. Cohen's *d*, Pearson's *r*), indicating how they were calculated

Our web collection on statistics for biologists contains articles on many of the points above.

Software and code

Policy information about availability of computer code

Data collection	Agilent Bravo VWorks 13.1.0
Data analysis	R v4.2.2<br>ggplot2<br>Python2<br>nf-core/eager v2.2.0<br>AdapterRemoval v2<br>Kraken 2<br>Burrows-Wheeler Aligner (BWA-0.7.17 aln)<br>SAMTools v1.3.1<br>DamageProfiler<br>BEDTools v2.29.2<br>Bamtools<br>FigTree<br>Q-TREE v.1.6.12<br>OxCal v4.4.4<br><br>https://github.com/pontussk/samremovedup<br>https://github.com/pontussk/mpileup_mismatch_pathogen.py

(https://github.com/pontussk/mpileup2consensus.py)

For manuscripts utilizing custom algorithms or software that are central to the research but not yet described in published literature, software must be made available to editors and reviewers. We strongly encourage code deposition in a community repository (e.g. GitHub). See the Nature Portfolio guidelines for submitting code & software for further information.

Data

Policy information about availability of data

All manuscripts must include a data availability statement. This statement should provide the following information, where applicable:

- Accession codes, unique identifiers, or web links for publicly available datasets
- A description of any restrictions on data availability
- For clinical datasets or third party data, please ensure that the statement adheres to our policy

All sequence data will be available in the European Nucleotide Archive upon publication. Fastq-formatted data of all non-human metagenomic sequences after processing via nf-core/Eager and excluding sequences mapping to hg37 human reference genome generated in this study have been deposited in the European Nucleotide Archive (ENA) at EMBL-EBI under accession number PRJEB61230 - https://www.ebi.ac.uk/ena/browser/view/PRJEB61230

Human research participants

Policy information about studies involving human research participants and Sex and Gender in Research.

Reporting on sex and gender	*Use the terms sex (biological attribute) and gender (shaped by social and cultural circumstances) carefully in order to avoid confusing both terms. Indicate if findings apply to only one sex or gender; describe whether sex and gender were considered in study design whether sex and/or gender was determined based on self-reporting or assigned and methods used. Provide in the source data disaggregated sex and gender data where this information has been collected, and consent has been obtained for sharing of individual-level data; provide overall numbers in this Reporting Summary. Please state if this information has not been collected. Report sex- and gender-based analyses where performed, justify reasons for lack of sex- and gender-based analysis.*
Population characteristics	*Describe the covariate-relevant population characteristics of the human research participants (e.g. age, genotypic information, past and current diagnosis and treatment categories). If you filled out the behavioural & social sciences study design questions and have nothing to add here, write "See above."*
Recruitment	*Describe how participants were recruited. Outline any potential self-selection bias or other biases that may be present and how these are likely to impact results.*
Ethics oversight	*Identify the organization(s) that approved the study protocol.*

Note that full information on the approval of the study protocol must also be provided in the manuscript.

Field-specific reporting

Please select the one below that is the best fit for your research. If you are not sure, read the appropriate sections before making your selection.

[x] Life sciences [ ] Behavioural & social sciences [ ] Ecological, evolutionary & environmental sciences

For a reference copy of the document with all sections, see nature.com/documents/nr-reporting-summary-flat.pdf

Life sciences study design

All studies must disclose on these points even when the disclosure is negative.

Sample size	No sample size calculations were made in this study, all specimens made available by archaeologists were sampled. This included 34 individuals from two sites, Charterhouse Warren and Levens Park.
Data exclusions	No data was excluded.
Replication	This study was on genome sequences retrieved from archaeological remains. No statistical replication was necessary.
Randomization	This study was on genome sequences retrieved from archaeological remains. No randomization was possible.
Blinding	Blinding was not applicable as it was unknown that the individuals had Yersinia pestis DNA.

Reporting for specific materials, systems and methods

We require information from authors about some types of materials, experimental systems and methods used in many studies. Here, indicate whether each material, system or method listed is relevant to your study. If you are not sure if a list item applies to your research, read the appropriate section before selecting a response.

Materials & experimental systems

n/a	Involved in the study
☒	☐ Antibodies
☒	☐ Eukaryotic cell lines
☐	☒ Palaeontology and archaeology
☒	☐ Animals and other organisms
☒	☐ Clinical data
☒	☐ Dual use research of concern

Methods

n/a	Involved in the study
☒	☐ ChIP-seq
☒	☐ Flow cytometry
☒	☐ MRI-based neuroimaging

Palaeontology and Archaeology

Specimen provenance

Charterhouse Warren samples, Somerset, UK are curated by the Wells and Mendips Museum. Sampling permission was given prior to sampling by Rick Schulting and the Museum on Wells and Mendips in April 2019.

Levens samples, Cumbria, UK are curated by the Levens Historical Group and sampling permission was given by Mr. Richard Bagot in Jan 2021.

Specimen deposition

Specimens remain curated by their respective institutions.

Dating methods

The mandible associated with one of the teeth (C10098) has been directly radiocarbon dated to 4,145-3,910 cal BP (95.4% confidence; OXA-37840: 3,685 ± 30 BP) by Oxford Radiocarbon Accelerator Unit and calibrated using IntCal20.
The Levens skeleton (C10928) has been radiocarbon dated to 4,229-3,976 cal BP (calibrated using IntCal20) (95% confidence, 3,731 ±34 BP, GU-51283).

☒ Tick this box to confirm that the raw and calibrated dates are available in the paper or in Supplementary Information.

Ethics oversight

Except the sampling permission provided, no further ethical approval or guidance was required as this study comprises only archaeological material older than 100 years, curated by individual institutions and researchers.

Note that full information on the approval of the study protocol must also be provided in the manuscript.
